# Optic nerve sheath meningioma detected by single- photon emission computed tomography/computed tomography somatostatin receptor scintigraphy: a case report

**DOI:** 10.1186/s13256-016-0885-8

**Published:** 2016-04-22

**Authors:** Lucie Nussbaum-Hermassi, Guido Ahle, Chistophe Zaenker, Camelia Duca, Izzie Jacques Namer

**Affiliations:** 1 Service de Biophysique et Médecine Nucléaire, Hôpital de Hautepierre, Hôpitaux Universitaires de Strasbourg, 1, avenue Molière, 67098 Strasbourg, Cedex 09 France; 2 Service de Neurologie, Hôpitaux Civils de Colmar, Colmar, France; 3 Service de Radiologie, Hôpitaux Civils de Colmar, Colmar, France; 4 ICube, Université de Strasbourg/CNRS (UMR 7357), Strasbourg, France; 5 Fédération de Médecine Translationnelle de Strasbourg (FMTS), Faculté de Médecine, Strasbourg, France

**Keywords:** Optic nerve sheath meningioma, SPECT/CT, Somatostatin receptor, Pentetreotide

## Abstract

**Background:**

Optic nerve sheath meningiomas account for only 2 % of orbital lesions and 42 % of optic nerve tumors. Diagnosis remains difficult because histologic confirmation carries a high risk of visual loss. Therefore, a less invasive and specific diagnostic method for differentiating optic nerve sheath meningiomas from other optic nerve lesions is needed to overcome the limitations of computed tomography and magnetic resonance imaging, and make the best individualized treatment decision. This case is a good illustration of the clinical and imaging difficulties inherent in this rare tumor, which may be hard to differentiate from other causes.

**Case presentation:**

A 51-year-old Caucasian woman developed a central scotoma, visual loss, and abnormal visual evoked potentials. The first magnetic resonance imaging scan classified the optic nerve damage as retrobulbar optic neuritis. After magnetic resonance imaging follow-up at 3 months, a negative lumbar puncture and biological workup, and clinical worsening, an optic nerve sheath meningioma was suspected. We confirmed this diagnosis with ^111^In-pentetreotide single-photon emission computed tomography, which is able to bind with very high affinity to somatostatin receptor subtype 2 expressed on meningiomas.

**Conclusions:**

In the diagnosis of optic nerve sheath meningiomas, [^111^In]-pentetreotide single-photon emission computed tomography-fused magnetic resonance imaging is a valuable additional tool, optimizing the diagnosis and obviating the need for a more invasive procedure.

## Background

Optic nerve sheath meningiomas (ONSMs) account for 2 % of orbital lesions and are the second most common optic nerve tumors, after optic nerve gliomas [1–4]. Clinically, optic nerve tumors closely mimic optic neuritis with painless unilateral vision impairment [2, 5]. Diagnosis remains difficult because histologic confirmation carries a high risk of visual loss. Therefore, a less invasive and specific diagnostic method for differentiating ONSMs from other optic nerve tumors is crucial for individualized treatment decisions. Meningiomas intensely express the somatostatin receptors [6–13], and receptor-negative cases are extremely rare [8]. Since somatostatin receptor scintigraphy has applications in the differential diagnosis between ONSMs and other orbital lesions, magnetic resonance imaging (MRI) is particularly valuable for evaluation of orbital neoplasms because it provides critical anatomic information on the ocular structures involved, perineural spread and intracranial extension [3].

We present a case of ONSM diagnosed using [^111^In]-pentetreotide single-photon emission computed tomography/computed tomography (SPECT/CT) in a patient initially followed for a diagnosis of optic neuritis.

## Case presentation

**Fig. 1:**
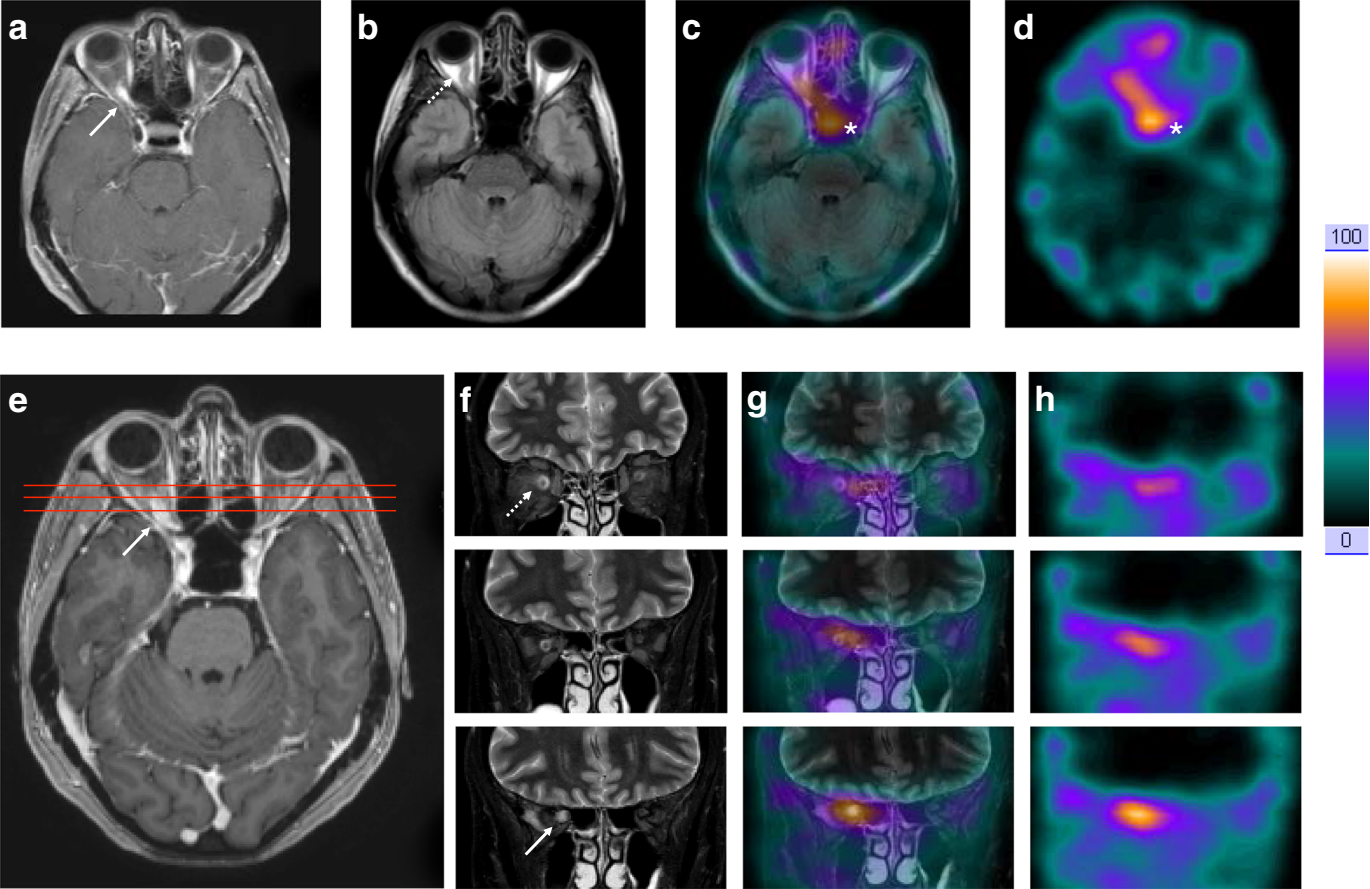
Multimodal imaging: matched transverse, postcontrast spin-echo T1-weighted (**a**), and fluid-attenuated inversion recovery (**b**) 1.5-T MRI scans, fused MRI/SPECT (**c**) and ^111^In-pentetreotide SPECT images (**d**). Transverse postcontrast gradient echo T1-weighted MRI scan (**e**) showing the location of coronal slices, and three-level coronal T2-weighted fat-suppressed MRI scan (**f**), fused MRI/SPECT image (**g**) and [^111^In]-pentetreotide SPECT image (**h**). MRI scan showed right tortuous optic nerve enlargement (**b**, *dotted arrow*), with a peripherally increased signal on fluid-attenuated inversion recovery (**b**, *dotted arrow*) and on coronal T2-weighted fat-suppressed sequences (**f**, *dotted arrow*). Therefore, uniform contrast enhancement (**a** and **e**, *solid arrows*) affected the last third of the nerve corresponding to an intense [^111^In]-pentetreotide uptake (**d** and **h**). The asterisk (*) shows the physiological uptake of [^111^In]-pentetreotide in the pituitary (**c** and **d**)

A 51-year-old Caucasian woman initially consulted for right visual loss (2/10, with corrective lenses) and central scotoma demonstrated with perimetry. Visual evoked potentials confirmed the altered conduction of the right optic nerve. In the absence of any other obvious neurologic symptoms and signs, optic neuritis was suspected. An initial MRI scan showed right tortuous optic nerve enlargement with a peripherally increased signal on fluid-attenuated inversion recovery (Fig. 1b) and on coronal T2-weighted fat-suppressed (Fig. 1f) sequences. Therefore, uniform contrast enhancement (Fig. 1a and e) affected the last third of the nerve.

Three months later, follow-up MRI demonstrated the persistent signal abnormalities on MRI. A comprehensive biological workup including lumbar puncture – initially refused by the patient – showed no abnormal results, and an ONSM was suspected.

SPECT/CT was performed 24 h after injection of 180 MBq [^111^In]-pentetreotide and showed an intense uptake in the last third of the intraorbital right optic nerve (Fig. 1d and h) corresponding to the contrast enhancement on the MRI scan. After this confirmation, she was referred to the radiotherapy center for stereotaxic radiotherapy. A treatment consisting in 30 sessions of stereotaxic radiotherapy was planned. Our patient received a total of 54 Gy by photons of 6 MeV (1.8 Gy per session), which was well tolerated. The 1-year clinical follow-up was positive with disappearance of the Marcus Gunn pupil sign, better visual acuity (7/10, with corrective lenses), and stability on MRI imaging.

### Discussion

Although meningiomas are common intracranial tumors, ONSMs as a specific subset are infrequent, accounting for only 1–2 % of all meningiomas [14]. Since morphological imaging techniques have their limitations in differentiating meningiomas from other tumors of the optic pathway [3, 6, 14, 15], an alternative method was added to detect whether this lesion expressed somatostatin receptors. Thus SPECT/CT or positron emission tomography/computed tomography (PET/CT) imaging with somatostatin analog radiotracers are highly effective methods for detection of meningiomas [6–13].

In accordance with a few studies on ONSMs [6, 7], the case reported herein showed an intense uptake of [^111^In]-pentetreotide in the last third of the intraorbital right optic nerve (Fig. 1c, d, g and h), as well as physiological pituitary uptake (Fig. 1c and d). This radiotracer presents a very high affinity for somatostatin receptor subtype 2 with high sensitivity and specificity, as well as [^111^In]-octreotide. There have also been reports on using somatostatin to treat intracranial meningiomas and monitor the efficacy of the treatment [16].

Consequently, this imaging technique provides good proof so that the exact origin of certain optic nerve tumors can be identified, especially and specifically in cases of very small tumors, in order to avoid biopsy and give the best treatment available quickly. In the majority of ONSMs involving the orbit, complete surgical resection is not possible and the results of surgical decompression are poor, despite the natural progression of diseases with progressive visual loss [15]. The diagnosis of ONSM is a crucial one, because modern surgical and/or radiotherapy approaches at an early stage may allow total resection and improve the chances of preserving vision [14, 15, 17–19]. For this reason, our patient could be treated with the best adapted procedure, resulting in better visual acuity. This technique with multimodal imaging provided positive identification rapidly, and our patient was spared more permanent visual damage or intracranial growth. A surgical approach was not adapted and presented a risk in this case due to the location and the small size.

This case supports other studies [6, 7] that have demonstrated a high [^111^In]-pentetreotide uptake by ONSMs, and shows the importance of making an early and noninvasive diagnosis compared to other orbital lesions, that is, optic nerve gliomas, optic nerve inflammation, non-Hodgkin lymphomas, vascular lesions, and sarcoidosis [2, 3]. In the present case, it was the most effective method to reverse the initial diagnosis of optic neuritis.

## Conclusions

In the diagnosis of ONSMs, somatostatin receptor subtype 2 detected with [^111^In]-pentetreotide SPECT/CT fused on MRI is a valuable additional tool, optimizing the diagnosis and obviating the need for a more invasive procedure.

## Consent

Written informed consent was obtained from the patient for publication of this case report and any accompanying images. A copy of the written consent is available for review by the Editor-in-Chief of this journal.

## Abbreviations

CT: computed tomography
MRI: magnetic resonance imaging
ONMS: optic nerve sheath meningiomas
PET/CT: positron emission tomography/computed tomography
SPECT/CT: single photon emission computed tomography/computed tomography
